# Complement is dispensable for neurodegeneration in Niemann-Pick disease type C

**DOI:** 10.1186/1742-2094-9-216

**Published:** 2012-09-17

**Authors:** Manuel E Lopez, Andres D Klein, Matthew P Scott

**Affiliations:** 1 Departments of Developmental Biology, Genetics, and Bioengineering, Howard Hughes Medical Institute, Stanford University School of Medicine, Clark Center W200, 318 Campus Drive, Stanford, CA, USA

**Keywords:** Complement, Neurodegeneration, Lysosomal storage disease, Niemann-Pick, Purkinje neurons, Microglia, Extracellular matrix

## Abstract

**Background:**

The immune system has been implicated in neurodegeneration during development and disease. In various studies, the absence of complement (that is, C1q deficiency) impeded the elimination of apoptotic neurons, allowing survival. In the genetic lysosomal storage disease Niemann-Pick C (NPC), caused by loss of NPC1 function, the expression of complement system components, C1q especially, is elevated in degenerating brain regions of *Npc1*^*-/-*^ mice. Here we test whether complement is mediating neurodegeneration in NPC disease.

**Findings:**

In normal mature mice, *C1q* mRNA was found in neurons, particularly cerebellar Purkinje neurons (PNs). In *Npc1*^*-/-*^ mice, *C1q* mRNA was additionally found in activated microglia, which accumulate during disease progression and PN loss. Interestingly, C1q was not enriched on or near degenerating neurons. Instead, C1q was concentrated in other brain regions, where it partially co-localized with a potential C1q inhibitor, chondroitin sulfate proteoglycan (CSPG). Genetic deletion of C1q, or of the downstream complement pathway component C3, did not significantly alter patterned neuron loss or disease progression. Deletion of other immune response factors, a Toll-like receptor, a matrix metalloprotease, or the apoptosis facilitator BIM, also failed to alter neuron loss.

**Conclusion:**

We conclude that complement is not involved in the death and clearance of neurons in NPC disease. This study supports a view of neuroinflammation as a secondary response with non-causal relationship to neuron injury in the disease. This disease model may prove useful for understanding the conditions in which complement and immunity do contribute to neurodegeneration in other disorders.

## Findings

### Introduction

Elevated immune and inflammatory factors are suspect in causing or promoting neurodegeneration in several neurological disorders. For the neurodegenerative lysosomal storage disease Niemann-Pick C (NPC), multiple independent gene profile studies analyzing *Npc1*^*-/-*^ mouse tissues and patient blood samples have identified immune response and inflammation pathway genes as the largest group whose expression is modified during disease progression [1]. Although these genes can be used as indicators of disease severity, the relevance of these inflammatory mediators to the pathology remains unclear. Previously, we observed that deletion of the inflammatory chemokine *Ccl3* gene did not have the beneficial effect on neurodegeneration in NPC-diseased mice that was evident for another lysosomal storage disorder, Sandhoff disease [2]. In addition to chemokines, complement components, Toll-like receptors, proteases, and apoptotic facilitators are also found to be elevated in the brains of *Npc1*^*-/-*^ mice. Components in these pathways could play critical roles in the disease progression. Here, we focus primarily on defining the role of the innate immune complement component C1q in NPC disease, since in other neurodegenerative scenarios C1q has been proposed to mediate synapse removal and mark apoptotic neurons for lysis and clearance [3-5].

### Expression and localization of C1q in mice with NPC disease

**Figure 1:**
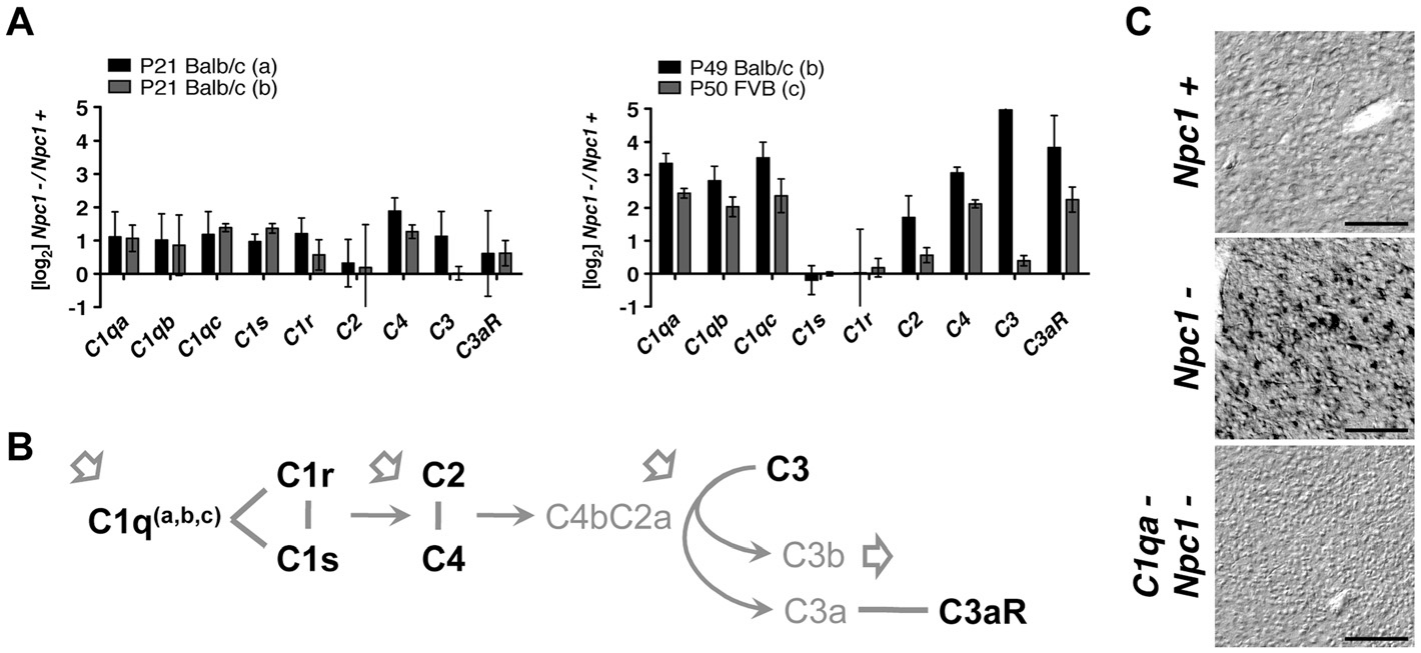
**C1q and complement component expression is increasingly elevated with age in NPC disease. **(**A**) Compilation of analyses from three independent cerebellar array datasets accessible through the National Center for Biotechnology Information (NCBI) Gene Expression Omnibus (GEO) database: a, GSE36119; b, GSE5944; and c, GSE20450. The differential gene expression of components of the complement cascade for *Npc1*^*-/-*^ mice compared to wild-type, *Npc1*^*+/-*^ or *Npc1*^*+/+*^, mice are shown at two ages. (**B**) An outline of the initial components of the complement cascade (genes are in bold) showing factors in the pathway that have been identified as elevated in at least one array dataset. (**C**) Representative *in situ *signal of *C1qa *mRNA probe hybridized to thalamic region of P60 wild-type, *Npc1*^*-/-*^, and *C1qa*^*-/-*^; *Npc1*^*-/-*^ mice. Scale bars are 100μm.

Using microarrays to analyze cerebellar mRNAs, independent studies using the *Npc1*^*-/-*^ NPC mouse model have found increased levels of complement genes, suggesting that complement may play an important role in the disease. The complement pathway involves serial processing of several protein complexes [4]. The classical complement cascade begins with C1q (C1qA, C1qB, and C1qC complex). Elevated levels of *C1qa*, *b*, and *c* mRNA have been detected in the cerebellum of *Npc1*^*-/-*^ mice as early as postnatal day 21 (P21), an age well before onset of severe neurodegeneration [6,7] (Figure 1A). At around P50, an age just prior to major decline in health, *C1qa*, *b*, and *c* mRNA levels are markedly higher [2,7]. In addition to the cerebellum, increased *C1qa* expression was found in the thalamus (Figure 1C), another brain region that is highly vulnerable in the disease. Downstream gene components of the complement cascade identified in cerebellar arrays include the anaphylatoxin and opsonin precursor C3 and the anaphylatoxin receptor C3aR. The mRNA of C3, which plays a central role in complement activation (Figure 1B), is not as robustly detected as C3aR (Figure 1A). The mRNAs of C1r and C1s, components needed to initiate the classical complement cascade, are also not consistently elevated between studies. However, even without efficient activation of the complement cascade, C1q alone may act as a recognition molecule that tags apoptotic cells to facilitate their clearance by phagocytes [5].

**Figure 2:**
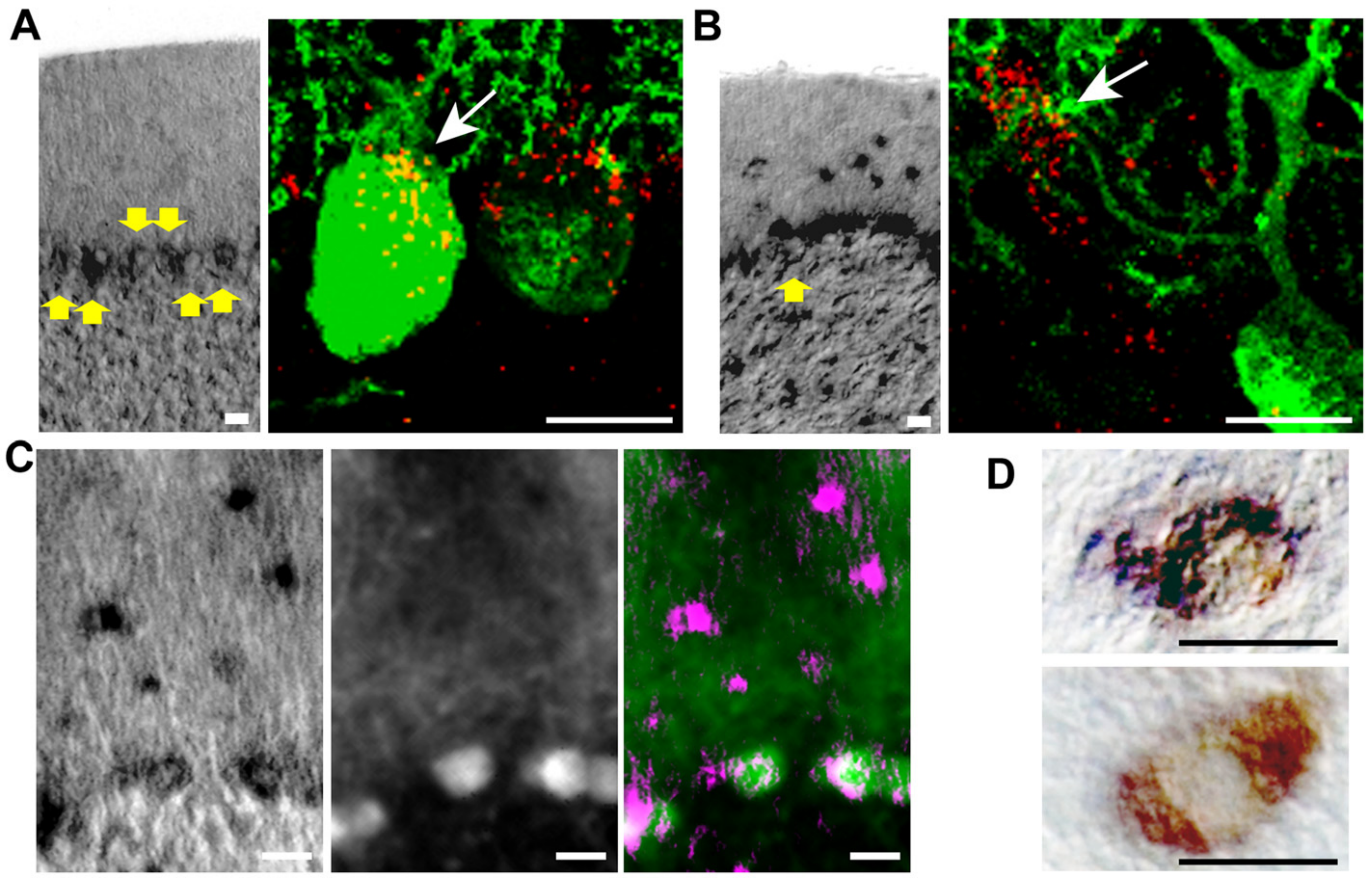
***C1qa *expression is induced in microglia in the brains of NPC mice. **(**A**) In wild-type mice, *C1qa *mRNA (left panel) and C1q protein (right panel) localize to cerebellar PNs (yellow arrows; anti-D28K green). A rendering of stacked confocal images (right panel) shows speckles of C1q (white arrow) internal to and proximal to the neuron. (**B**) In *Npc1*^*-/-*^ mice, *C1qa *mRNA (left panel) and C1q protein (right panel) do not localize entirely to PNs. (**C**) Overlay (right panel) of *C1qa *mRNA (left panel, magenta) with anti-D28K (middle panel, green). (**D**, top panel) NBT/BCIP reaction from *C1qa *RNA *in situ *hybridization (dark purple) co-localizes with DAB anti-CD68 immunohistochemistry (brown) in *Npc1*^*-/-*^ mice. (**D**, bottom panel) In *C1qa*^*-/-*^; *Npc1*^*-/-*^ mice, only the DAB staining can be seen. Scale bars are 10μm.

**Figure 3:**
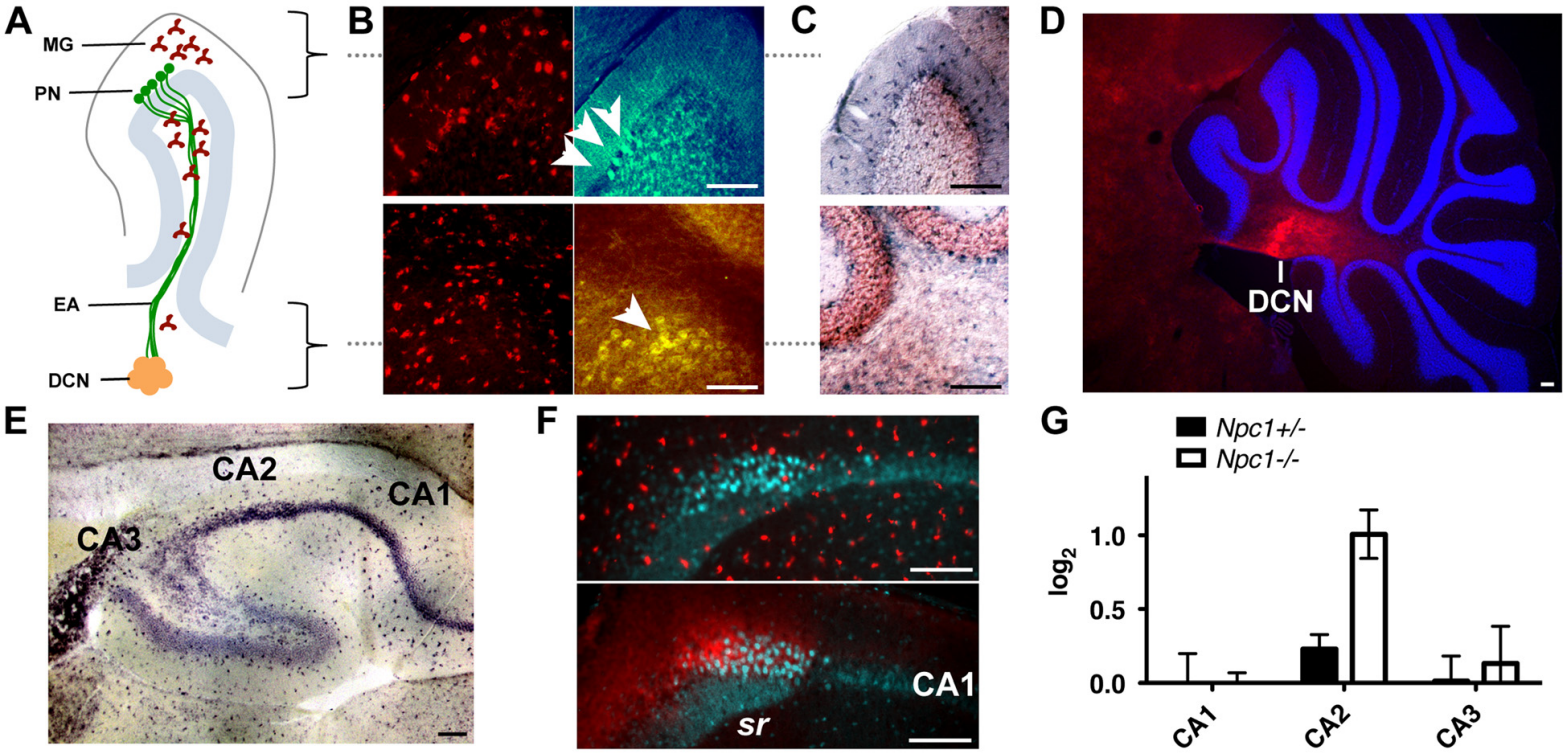
**C1q does not preferentially accumulate in brain regions with degenerating neurons in NPC mice. **(**A**) A simplified diagram of a cerebellar lobule marks the location of activated microglia (MG), degenerating Purkinje neurons (PN), and their efferent axons (EA) that contact the deep cerbellar nuclei (DCN) at the center of the cerebellum. (**B**, top panel) In the cerebellar cortex from an *Npc1*^*-/-*^ mouse, abundant microglia (anti-CD68, red) and few remaining PNs (Nissl stain, green; arrows) can be seen. (B, bottom panel) Microglia are also present throughout the cerebellum, though visibly less swollen, near remaining DCN neuron bodies (Nissl stain, yellow; arrow). (**C**) *C1qa *mRNA can be detected throughout the cerebellum. (**D**) Nevertheless, C1q protein (red) is found concentrated at the DCN in *Npc1*^*-/-*^ mice. Hoechst nuclear stain (blue) demarcates the cerebellar lobes. (**E**) Similarly, *C1qa *mRNA (dark purple) can be detected throughout the hippocampus, in microglia and neurons in *Npc1*^*-/-*^ mice. (**F**, top panel) Microglia (anti-CD68, red) evenly decorate the CA3, CA2, and CA1 neuron fields (anti-D28K, cyan), (F, bottom panel) however C1q protein (red) concentrates at the CA2 region (bright cyan) opposite the stratum radiatum (sr) side of the CA3/CA2 field. (**G**) Analysis of the C1q immunofluorescence intensity shows a peak difference between CA1 and CA2. Graph depicts the mean log fold change and 95% confidence limit of fluorescent intensity between *Npc1*^*-/-*^ and *C1qa*^*-/-*^; *Npc1*^*-/-*^ mice compared to *Npc1*^*+/-*^ and *C1qa*^*-/-*^; *Npc1*^*-/-*^ mice. The average log fold change of C1q immunofluorescence in *C1qa*^*-/-*^; *Npc1*^*-/-*^ hippocampal neuron regions over tissue autofluorescence is 0.15 with a standard deviation of 0.15. All images are representative of data observed in at least six mice at various ages >P50. Scale bars are 100μm.

We next determined if C1q, a secreted molecule, localizes around degenerating neurons in the NPC mouse model. In other neurodegenerative disease models, such as amyotrophic lateral sclerosis [8] and glaucoma [3], C1q is produced in and localizes around injured neurons. In normal mice, we detected *C1qa* mRNA in cerebellar Purkinje neurons (PNs) (Figure 2A). Antibody staining showed that C1q co-localized with PN soma, marked by Calbindin-D28K immunofluorescence. In *Npc1*^*-/-*^ mice, more abundant *C1qa* was detected in the cerebellum, but the additional mRNA was not produced by the degenerating PNs (Figure 2B, 2C). Instead, the majority of the *C1qa* mRNA co-localized with CD68, a marker for activated microglia, which was detected using 3,3^′^-diaminobenzidine tetrahydrochloride (DAB) immunohistochemistry (Figure 2D). Strikingly, C1q protein was not enriched in areas of neuron loss or high microglia activity such as the cerebellar cortex, but was instead concentrated in specific brain regions, for example, the deep cerebellar nuclei (DCN) of the cerebellum (Figure 3A-D) and the CA2 region of the hippocampus (Figure 3E-G). Although many efferent axons of degenerating neurons contact the DCN and CA2, DCN and CA2 neurons are not extraordinarily vulnerable to NPC-triggered degeneration [9]. Thus, in contrast to other neurodegenerative conditions [3,8,10], C1q does not preferentially mark degenerating neurons in NPC disease.

**Figure 4:**
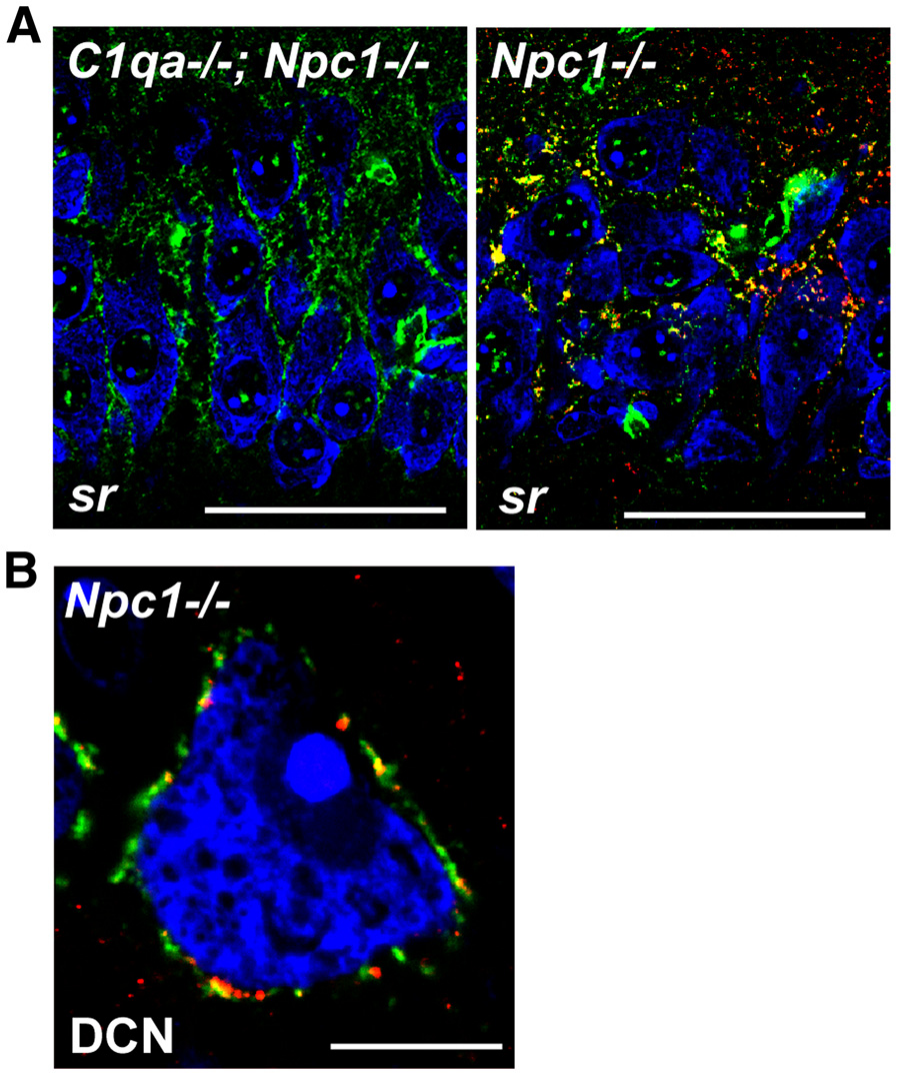
**C1q localizes to the perineuronal ECM in the CA2 hippocampus and cerebellar DCN. **(**A**) In *Npc1*^*-/-*^ mice, C1q (red) partially localizes with CSPG (green) of CA2 neurons. Matched image from a *C1qa*^*-/-*^; *Npc1*^*-/-*^ mouse is shown as a control for the C1q antibody used. Sr denotes the stratum radiatum side of the CA2 field. CA2 neuron bodies (blue) are marked with Nissl stain. Scale bars are 50μm. (**B**) A single cerebellar DCN neuron is shown. Scale bar is 10μm.

It is possible that the extracellular matrix may precipitate C1q and arrest its activity during NPC disease progression. DCN and CA2 neurons possess an extensive perineuronal extracellular matrix (ECM) composition [11,12]. In the DCN and CA2, C1q partially co-localized with the ECM component chondroitin sulfate proteoglycan (CSPG) (Figure 4A, 4B). Secreted CSPGs are known to strongly bind C1q functional domains, which may prevent C1q binding to the cell surface, inhibit C1q complex formation with C1r and C1s, and/or interfere with C1q binding to its receptor on phagocytes [13]. Thus, C1q found around DCN and CA2 neurons may be sequestered there and inactivated by perineuronal CSPG. How C1q localizes to specific brain regions, whether these regions are mainly efferent axonal connections of degenerating neurons, and whether C1q serves a function at these sites remains to be investigated.

### NPC-induced patterned neurodegeneration continues despite genetic inactivation of complement

**Figure 5:**
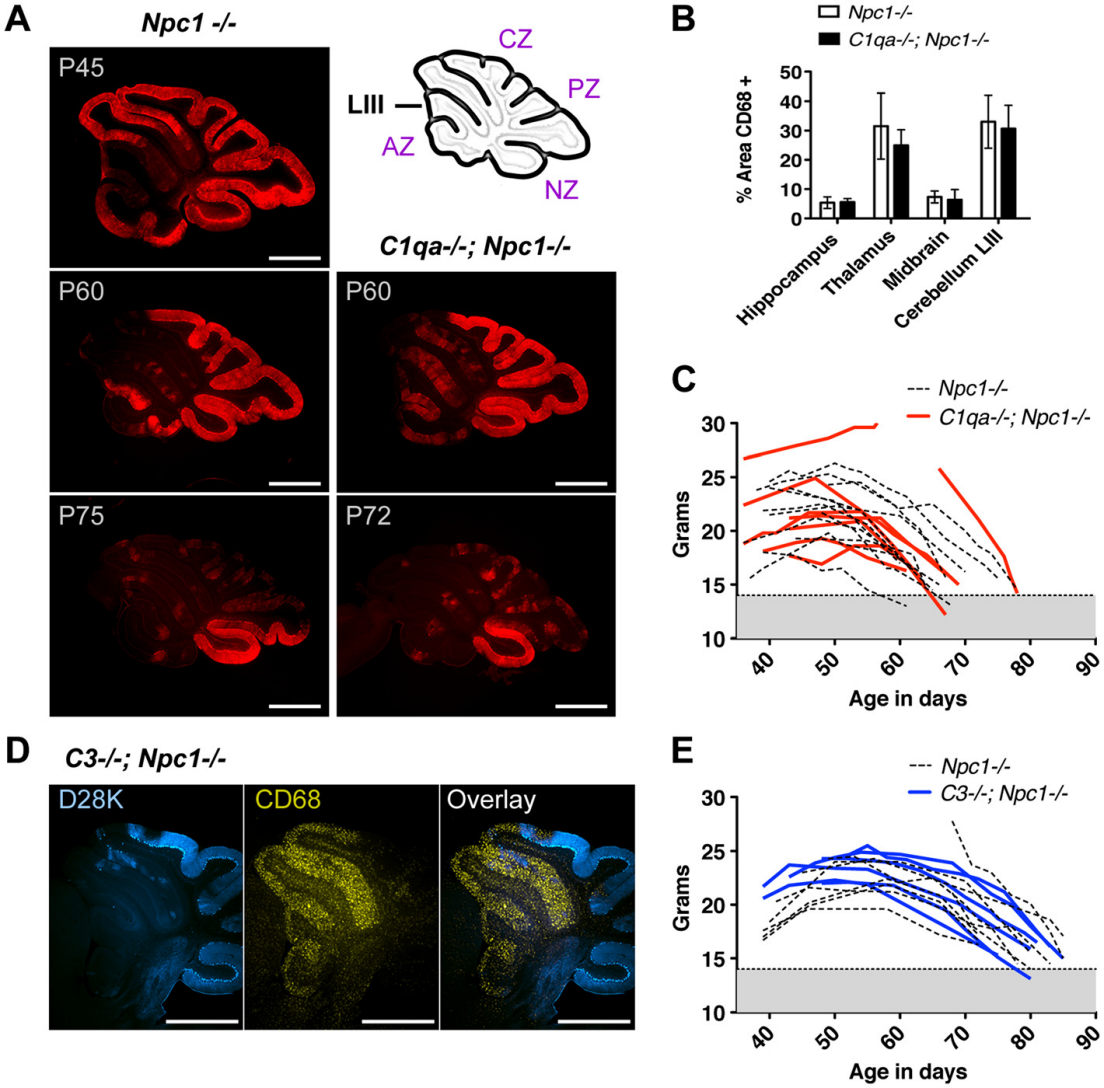
**Complement deficiency does not affect patterned Purkinje neuron death and disease progression. **(**A**) Cerebellar PNs (anti-D28K, red) in the anterior zone (AZ) die before PNs in the central and posterior zone (CZ and PZ), and PNs in the nodular zone (NZ) survive regardless of age. This patterned PN loss was not altered by C1q deficiency. (**B**) C1q deficiency also did not change the level of activity of microglia cells, marked by anti-CD68 and quantified by percent area occupied in the tissue, throughout the brain. The cerebellar cortex of lobule III was used to determine the level of microglia activity at P60. (**D**) Patterned PN (anti-D28K, blue) loss and the inverse pattern of microglial (anti-CD68, yellow) activity was also detected in *C3*^*-/-*^; *Npc1*^*-/-*^ mice. (**C**, **E**) Two-way ANOVA analysis on the weight profiles of *Npc1*^*-/-*^ mice showed that neither *C1qa *(red lines, P = 0.70) nor *C3 *(blue lines, P = 0.41) deficiency significantly affected disease progression. Non-*C1qa *or *C3 *deficient *Npc1*^*-/-*^ sibling mice were used for comparison (dashed lines).

A common observation in genetic neurodegenerative disorders is selective neuron vulnerability to ubiquitous toxic factors [14]. In NPC disease, selective vulnerability is easily traceable in the cerebellum where PNs, one of the more susceptible neurons to NPC1 deficiency, undergo a highly organized patterned loss [15,16]. The sequence of PN loss across cerebellar lobules (Figure 5A) provides a reliable phenotype for judging the extent of neuron rescue after a manipulation. As a foundation for the present analysis, we have previously shown that PN-specific production of NPC1 prevents patterned neuron loss in otherwise *Npc1*^*-/-*^ mice [17]. Here, we assessed whether deletion of *C1qa* or *C3* genes [3] could modify PN loss. We found that patterned PN loss was not notably altered in the cerebellum. Purkinje neurons continued to degenerate throughout cerebellar lobular zones (Figure 5A, D). Microglial activity and weight analysis [17] were further used to demonstrate the absence of significant changes in overall disease progression (Figure 5B, C-E). We conclude that, in this mouse model of NPC disease, complement is not required to mediate neurodegeneration and subsequent neuroinflammation.

### Loss of other immune-related components, or an apoptosis facilitator, does not alter patterned neuron loss in NPC mice

Our prior experiments have demonstrated that neuron death and survival in NPC disease is driven by more dominant cell-autonomous mechanisms [2,9,17], and we suggested that immune factors might have minimal involvement. The continued patterned neuron loss in NPC disease, even with loss of complement, supports these conclusions. However, many other immune and inflammatory factors may be more heavily involved in mediating neurodegeneration. Using existing mutant mice, we have sampled a few other genes that are substantially over-expressed in NPC disease based upon reported datasets [1,2,6,7]. Loss of function of TLR7, an endosomal Toll-like receptor involved in innate immunity [18], UNC93b1, an associated mediator of TLR activity [19], MMP12, an extracellular matrix macrophage metalloprotease [20], and the pro-apoptotic BH3-only Bcl-2 family member BCL2L11, also known as BIM, [21,22] individually did not alter the patterned PN loss in *Npc1*^*-/-*^ mice (Table 1). These results demonstrate that, despite the evident presence of immune response and inflammatory mediators, neurons will die in their typical NPC manner with or without these factors.

**Table 1:** Deficiency in other immune response-relevant pathways does not alter neurodegeneration in NPC disease

***Npc1***^***-/-***^***& ____***	**Function/Pathway**	**Mice**^**a**^**(*n*)**	**Change in PN loss**	**Change in microglia**	**Change in %CD68**^**b**^	**Ref.**
*C1qa*^*-/-*^	Complement	11	No	No	*P* = 0.49	Figure 5B
*C3*^*-/-*^	Complement	7	No	No	*P* = 0.88	
*Mmp12*^*-/-*^	ECM degradation	4	No	No	*P* = 0.95	
*Bcl2l11(Bim)*^*-/-*^	Apoptosis	4	No	No	*P* = 0.17	
*Tlr7*^*-/-*^	Toll-like receptor	3	No	No	n/a	
*Unc93b1*^*3d/3d*^	Toll-like receptor	3	No	No	n/a	
*Pcp2-tTA; TetO-Npc1YFP*	Purkinje neuron (PN) rescue	32	Yes	Yes	*P* = 0.0057	[2,17]

^a^Number of mice used to observe PN loss and microglial activity for ages >P60, see Figure 5A and 5D.

^b^Two-way ANOVA: Does the gene affect %CD68 in the cerebellum or thalamus of *Npc1*^*-/-*^ mice compared to *Npc1*^*-/-*^ controls? *P* value <0.05 is considered significant; *n* = 4 for all mice at age P60. n/a means a statistical analysis was not performed for this group. Hippocampus and midbrain values, which showed no difference across samples, were used to normalize %CD68 in thalamus and cerebellum, respectively. Note: PN rescue corrects cerebellar but not thalamic pathology [17], *P* = 0.0005.

### General conclusions

In this study, we show that C1q and other immune factors do not facilitate the elimination of dying neurons in the disease. This study agrees with a commonly employed mouse model of Parkinson disease where microglial C1q is reported to not affect nigrostriatal dopaminergic injury [23]. The cell-autonomous and genetically controlled neuron survival in the NPC mouse model [2,17] provides a tool and opportunity for uncovering alternate non-apoptotic mechanisms of neuron death and clearance that may also occur in many other neurodegenerative disorders.

## Methods

Mice were managed in accordance with Stanford University’s Administrative Panel on Laboratory Animal Care. *Npc1*^*+/-*^ mice were derived as previously reported [17] and crossed with *C1qa*[24] or *C3*[25] knockout mice obtained at Stanford University [3]. *Tlr7*[18], *Mmp12*[20], and *Bcl2l11(Bim)*[21] knockout mice were obtained from the Jackson Laboratory. *Unc93b1*^*3d*^[19] mutant mice were obtained from the Mutant Mouse Regional Resource Centers. Genotyping was performed as detailed in the references listed above. To minimize background discrepancies, sibling offspring were used for comparisons. For example, mixed FVB/B6 *C1qa*^*+/-*^; *Npc1*^*+/-*^ mice were mated to produce offspring with genotypes *Npc1*^*-/-*^; *C1qa*^*+/+*^ and *Npc1*^*-/-*^; *C1qa*^*-/-*^. Isolated brains were fixed whole overnight at 4°C in 4% paraformaldehyde in phosphate buffer saline (PBS).

Immunofluorescent and imaging procedures were performed as previously described [17]. Primary antibody sources are as follows: rat anti-C1q (Abcam), rat anti-CD68 (AbdSerotec), rabbit and mouse anti-Calbindin-D28K (Sigma), and mouse anti-CSPG (Millipore). Stainings include Hoechst (Invitrogen) and NeuroTrace 435/455 fluorescent Nissl stain (Invitrogen). ImageJ and GraphPad Prism software were used for measurements and statistics. Unless stated otherwise, means and standard deviations are reported.

DIG-labeled antisense probes for *in situ* detection of *C1qa* were designed as previously described [3]. A total of 200 ng/mL of purified DIG-labeled RNA probe was hybridized to 50 μm thick vibratome tissue sections that were processed in RNAse-free 5x SSC buffer. Hybridization was performed at 60°C for 16 h in hybridization solution: 50% formamide, 5x SSC, 0.1% Tween-20, 500 ug/mL tRNA, 500ug/mL salmon sperm DNA, 50 ug/mL Heparin salt, and 0.5% SDS. Washes were done at 60°C for 15 min using hybridization buffer followed by 5x SCC and 0.2x SSC buffers with 0.1% Tween-20 (Sigma). Afterwards, PBS with 2% BSA and 0.2% Triton X-100 (Sigma) was used for incubating anti-Digoxigenin-AP, Fab fragments (Roche) overnight at 4°C. The NBT/BCIP or HNPP Fast Red reaction was then performed as commercially directed (Roche). To mark CD68-positive cells microglia with DAB (Sigma), 50 μm vibratome sections were first treated with 0.6% hydrogen peroxide in methanol for 30 min followed by incubation of anti-CD68 antibody in PBS with 2% BSA and 0.2% Triton X-100. Diethylpyrocarbonate (DEPC; Sigma) was present in 0.01% vol/vol concentration throughout the CD68 immunohistochemical procedure. Unlike anti-CD68, anti-D28K can be used after *in situ* hybridization for immunofluorescent detection of PNs.

## Competing interests

The authors declare that they have no competing interests.

## Authors’ contributions

MEL designed and performed the experiments, analyzed the data, and drafted the manuscript. ADK contributed to the analysis of C1q and C3 deficient mice. MPS collaborated in discussing the results and writing the manuscript. All authors read and approved the final manuscript.
